# The relationship between obesity and quality of life in Brazilian adults

**DOI:** 10.3389/fpsyg.2015.00966

**Published:** 2015-07-14

**Authors:** Fernanda B. C. Pimenta, Elodie Bertrand, Daniel C. Mograbi, Helene Shinohara, J. Landeira-Fernandez

**Affiliations:** Departamento de Psicologia, Pontifícia Universidade Católica do Rio de Janeiro, Rio de Janeiro, Brazil

**Keywords:** obesity, adults, quality of life, BMI, World Health Organization Quality of Life

## Abstract

The incidence of obesity has reached epidemic proportions, affecting 30% of the adult population globally. During the last decade, the rising rates of obesity in developing countries has been particularly striking. One potential consequence of obesity is a decline in quality of life (QoL). Thus, the objective of the present study was to investigate the possible relationship between obesity, defined by body mass index (BMI), and QoL, evaluated using the short version of the World Health Organization Quality of Life (WHOQOL) scale in a Brazilian population. The sample consisted of 30 men and 30 women, divided into three groups according to BMI: normal weight, obese, and morbidly obese. All of the subjects responded to the WHOQOL inventories. The results indicated that the groups with lower BMIs had better QoL than the groups with higher BMIs. Being overweight interfered with QoL equally in both sexes, with no difference found between men and women. The results indicate the necessity of multidisciplinary care of obese individuals.

## Introduction

Obesity is characterized by the excessive accumulation of body fat relative to lean mass. Its prevalence reached important proportions (Ng et al., 2014), being one of the main problems that face public health in modern societies (Halpern and Mancini, 2003). During the last decade, studies showed that obesity has increased in developing countries (Friedrich, 2002; Popkin et al., 2012). Obesity increases the risk of several chronic diseases, such as diabetes mellitus, cardiovascular and cerebrovascular disease, coagulation alterations, degenerative articulation disease, neoplasias, and sleep apnea, among others (Dixon, 2010; Vucenik and Stains, 2012; Ojeda et al., 2014). Regarding the relationship between obesity and mortality, a recent study showed an increased risk only for patients with a higher grade of obesity (Flegal et al., 2013). However, most of the results published highlighted that obese patients have an increase in mortality (Must et al., 1999; Adams et al., 2006; Guh et al., 2009).

The most commonly used method to assess obesity in adults is the body mass index (BMI). BMI is calculated as body weight (in kilograms) divided by the square of height (in meters). BMI is highly correlated with body fat, but it does not directly measure the proportion of fat. Table 1 presents the classification of BMIs according to the World Health Organization. Subjects are considered obese, which is implicated in functional and health deficits, when they present a BMI between 30 and 40 kg/m^2^, a range that varies only in the degree of illness.

**TABLE 1:** **Weight ranges according to body mass index (BMI)**.

**Category**	**BMI**
Underweight	<18.5
Normal weight	18.5–24.9
Overweight	25.0–29.9
Obesity I	30.0–34.9
Obesity II	35.0–39.9
Obesity III	≥40.0

Obesity is considered as a multifactorial condition, implicating medical, psychiatric, and social aspects. According to Dobrow et al. (2002), obesity is a behavioral disorder that reflects excess food intake compared with energy expenditure; therefore, the genetic contribution to the initiation and maintenance of obesity needs to be considered.

The genetic predisposition to obesity can be expressed in different degrees, either higher or lower, but environmental factors also play a role (Silventoinen et al., 2010; Dubois et al., 2012; Blakemore and Buxton, 2014). The biological/environmental model rather than the psychological model best explains the behaviors that lead to obesity, although its consequences affect psychological variables. According to Dobrow et al. (2002), several studies have revealed high correlations between obesity, depression, and low self-esteem.

Regardless of the specific causes of obesity, its psychological aspects are important when considering the quality of life (QoL) of obese individuals. Studies showed that obese individuals are negatively impacted by judgments and direct and indirect criticisms from others (for review, see Puhl and Heuer, 2009). Thus, negative feelings about oneself and the world often occur, causing anxiety and depression. Negative personal beliefs about inferiority are common in obese individuals (Abilés et al., 2010; Luppino et al., 2010; Son and Kim, 2012). Additionally, excess body weight can cause muscle pain, articulation pain, and discomfort. Because the obese body is heavy and bulky, it expends more energy to move, consequently resulting in the need to rest more often.

The World Health Organization defines QoL as an individual’s perception of his or her position in life within the context of the culture and value systems in which he or she lives and relative to his or her objectives, expectancies, patterns, and preoccupations. QoL encompasses (1) physical aspects, such as pain, fatigue, energy, sleep, and rest, (2) psychological aspects, such as self-esteem, memory, positive and negative feelings, and perceptions of body image and appearance, (3) social aspects that principally regard personal relationships, and (4) environmental aspects, such as security, finances, leisure, and information (World Health Organization, 1996; Table 2). Studies on obesity and QoL suggest a possible interaction between these two variables (Hlatky et al., 2010; Buttitta et al., 2014; McLaughlin and Hinyard, 2014), in which physical, medical, and cultural aspects that are related to obesity are directly reflected by scores on evaluations of QoL. Illnesses associated with being overweight, the difficulties and embarrassment that obese individuals often experience, and cultural beliefs about beauty, functionality, productivity, and personality attributes (e.g., self-control and perseverance) can negatively interfere with the way of life of obese people.

**TABLE 2:** **Sample items of the World Health Organization Quality of Life-Brief (WHOQOL-Brief)**.

**Domains**	**Sample items**
Domain 1:	Do you have enough energy for everyday life?
physical health	How satisfied are you with your sleep?
Domain 2:	How much do you enjoy life?
psychological	How satisfied are you with yourself?
Domain 3:	How satisfied are you with your personal relationships?
social relationships	How satisfied are you with the support you get from your friends?
Domain 4:	How safe do you feel in your daily life?
environment	To what extent do you have the opportunity for leisure activities?

Even with some authors suggesting an influence of the cultural and ethnic context (Ritenbaugh, 1982; Perez and Warren, 2011; Cox et al., 2011), most studies demonstrating a relationship between QoL and obesity were conducted with North American and European populations. The few studies exploring this relationship in developing countries show contradictory results regarding the association between obesity and impaired QoL (e.g., Lee et al., 2012 in Malaysia; Boodai and Reilly, 2013 in Kuwait; Jalali-Farahani et al., 2013 in Iran). The discrepancy in findings was explained due to cultural differences in attitudes and stigma toward obesity, differences of social expectations for body size and differences regarding social norms. The few studies exploring the relationship between BMI and QoL in Brazil also had contradictory results. Adolescents (Turco et al., 2013) and women (Horta et al., 2013) with chronic non-communicable diseases experienced a similar impact of weight on QoL to samples in developing countries (QoL was negatively influenced by higher BMI). However, an interesting cross-cultural study involving Brazilian and Austrian women with Polycystic Ovary Syndrome demonstrated that for the Brazilian group, obesity was the factor having less impact on QoL, in contrast to the Austrian group, highlighting an influence of the cultural context on the obesity/QoL relationship (Hashimoto et al., 2003). The results of these studies show that the impact of weight on QoL is still unclear for the Brazilian population. Moreover, the specificity of the groups studied does not allow generalizing the results, highlighting the need for further exploration of the impact of BMI on QoL in the Brazilian population.

Gender has been shown to be another factor influencing the QoL burden of obesity. The existing literature indicates that the QoL of women and men is differentially impacted by excessive body weight, with a more impaired QoL in women than in men (Jia and Lubetkin, 2005; Bentley et al., 2011; Korhonen et al., 2014). Choo et al. (2014) showed similar results in a Korean population and suggested that sociocultural influences explained these findings. In fact, in various societies worldwide thinness is valued, resulting in pressure for individuals to monitor their body weight (for review see, Hesse-Biber et al., 2006).

The main objective of the present study was to investigate for the first time in an adult Brazilian population the relationship between QoL and obesity, testing the hypothesis that obesity would be associated with impaired QoL in this population differently in comparison to the results obtained in developed countries. The study also investigated the possible effect of gender on the relationship between obesity and QoL, testing the hypothesis that the influence of weight on QoL would differ between men and women.

## Materials and Methods

### Subjects

Sixty subjects, aged 20–60 years, were included in the study and divided into three groups (*n* = 20 per group; 10 men and 10 women) that were matched by age.

Weight and height were self-reported and used to calculate BMI, using the standard BMI formula of weight in kilos divided by height squared in meters (kg/height^2^). Subjects with BMIs between 18.5 and 24.9, which is considered normal, were included in the first group. The second group was composed of subjects who presented BMIs between 30.0 and 39.9, which are considered degrees I and II of obesity. The third group was composed of subjects with a BMI above 40.0, which is considered morbid obesity.

### Tools

To evaluate the QoL for each participant, the World Health Organization Quality of Life-Brief (WHOQOL-Brief) instrument was used, which was translated into Portuguese and validated for the Brazilian population (Fleck et al., 2000). The WHOQOL-Brief consists of 26 questions (two general questions that evaluate QoL and 24 questions that represent specific items in the physical, psychological, social, and environmental domains). This instrument also has a cover page that collects general information about the participant, such as age, sex, weight and height (to calculate the BMI), and health problems.

### Procedure

The participants were recruited at the Service of Applied Psychology of the Department of Psychology at PUC-Rio.

All subjects were informed about data confidentiality and anonymity before they responded to the questionnaire. The questionnaires were individually applied, and any doubts or questions about the study were clarified by the researcher.

### Statistical Analysis

Group differences in the total scores on the WHOQOL-Brief and each of the domains were analyzed using two-way analysis of variance (ANOVA; 3 × 2). The first factor was BMI (normal, obese, or morbidly obese), and the second factor was gender (male or female). The focus of interest in the analysis is the comparison between weight categories (main effect of BMI), with the gender × weight interactions being secondary interests. The same analysis was performed for the number of illnesses reported by the subjects. In both analyses, significant results were followed up with *post hoc* comparisons using Student’s *t*-test.

### Ethical Consideration

All participants provided informed consent before taking part in the study. The study was approved by the Ethical Review Board Committee from Pontifícia Universidade Católica from Rio de Janeiro and was conducted in accordance with the Declaration of Helsinki.

## Results

**FIGURE 1:**
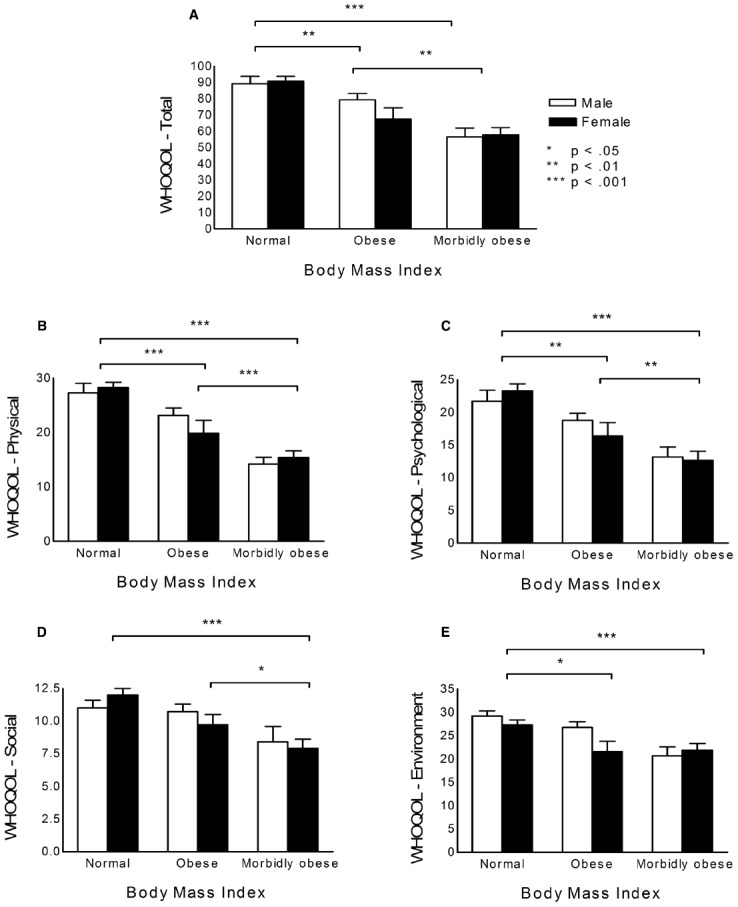
**Quality of life according to BMI ranges and gender. (A)** WHOQOL total scores. **(B)** Physical domain. **(C)** Psychological domain. **(D)** Social domain. **(E)** Environment domain.

Figure 1 presents the mean (±SEM) of the total score and score on each domain of the WHOQOL in men and women with normal weight, obesity, and morbid obesity.

The results were similar for the total scores and domain scores, revealing a main effect of BMI (total: *F*_2,54_ = 22.76, *p* < 0.001; physical: *F*_2,54_ = 35.22, *p* < 0.001; psychological: *F*_2,54_ = 19.93, *p* < 0.001; social: *F*_2,54_ = 9.70, *p* < 0.001; environmental: *F*_2,54_ = 10.24, *p* < 0.001), but no effect of gender (total: *F*_1,54_ = 0.53, *p* = 0.469; physical: *F*_1,54_ = 0.07, *p* = 0.793; psychological: *F*_1,54_ = 0.12, *p* = 0.727; social: *F*_1,54_ = 0.07, *p* = 0.791; environmental: *F*_1,54_ = 2.43, *p* = 0.124), and no interaction between factors (total: *F*_2,54_ = 1.25, *p* = 0.294, physical: *F*_2,54_ = 1.29, *p* = 0.285; psychological: *F*_2,54_ = 0.87, *p* = 0.423; social: *F*_2,54_ = 0.92, *p* = 0.404; environmental: *F*_2,54_ = 2.15, *p* = 0.126).

For total scores and physical and psychological domain scores, the *post hoc* comparisons revealed that the participants with normal weight displayed higher QoL compared with obese individuals (total: *p* = 0.001; physical: *p* < 0.001; psychological: *p* = 0.002) and morbidly obese individuals (total: *p* < 0.001; physical: *p* < 0.001; psychological: *p* < 0.001), and obese individuals displayed higher QoL compared with morbidly obese individuals (total: *p* = 0.001; physical: *p* < 0.001; psychological: *p* = 0.003). For the social domain, morbidly obese individuals displayed lower QoL compared with normal weight individuals (*p* < 0.001) and obese individuals (*p* = 0.010), but the latter two groups did not differ from each other (*p* = 0.096). For the environmental domain, individuals with normal weight displayed better QoL compared with obese individuals (*p* = 0.011) and morbidly obese individuals (*p* < 0.001). The latter two groups did not differ from each other (*p* = 0.065). These results are shown in Figure 1.

The ANOVA for number of illnesses revealed significant effects of BMI (*F*_2,54_ = 12.19, *p* < 0.001) and gender (*F*_1,54_ = 4.57, *p* = 0.037) but no interaction between these factors (*F*_2,54_ = 0.30, *p* = 0.740). The *post hoc* comparisons indicated a greater number of illnesses in women than in men (*p* = 0.037) and a smaller number of illnesses in individuals with normal weight compared with obese (*p* = 0.002) and morbidly obese (*p* < 0.001) individuals.

## Discussion

The results indicated an association between an increase in BMI and a decrease in QoL in all domains of the WHOQOL-Brief. For total scores and scores on the physical and psychological domains, the results suggested a linear reduction of QoL with an increase in BMI. The analysis also suggested an increase in the number of comorbidities with an increase in BMI.

The findings with global scores on the WHOQOL-Brief supported previous studies that reported a negative linear relationship between QoL and elevated BMI (Kushner and Foster, 2000; Taylor et al., 2013; Ul-Haq et al., 2013; for review, see Kolotkin et al., 2001). However, these results are in contrast with our hypothesis of cultural differences. These findings can be explained by recent changes observed regarding body image in Brazil. In fact, esthetic ideals in the Brazilian society have been through a progressive transition from a curvier to a thinner body shape, closer to the beauty ideal of European and North American societies (Goldenberg, 2010; Forbes et al., 2012). Following these changes regarding beauty ideal, the stigma toward obesity increased, with greater valorization of thinness (Oliveira and Hutz, 2010). This sociocultural context can explain the negative impact of BMI on QoL.

In contrast to previous studies (Garner et al., 2012; Choo et al., 2014; Korhonen et al., 2014), there was no effect of gender on this relationship. This discrepancy may be explained by sociocultural differences, such as the specific values of the Brazilian culture regarding body image, given that the role of these contextual variables has already been suggested in the relationships among gender, BMI, and QoL (Choo et al., 2014). Studies in the last decade highlighted that Brazilian men are also subject of a sociocultural pressure regarding body image (Oliveira and Hutz, 2010). Kakeshita and Almeida (2008) highlighted the impact of this form of social pressure on men, indicating that both men and women were dissatisfied with their body size and desired leaner bodies.

The results with regard to the different domains also indicated a relationship between elevated BMI and poor QoL. The physical aspect of QoL was negatively correlated with BMI. This domain considers such characteristics as pain, sleep, and the capacity to perform daily activities. Stone and Broderick (2012) reported that BMI and pain were positively correlated, and others reported an association between obesity and sleep disorders (Lam et al., 2012; Ryan et al., 2014). In a recent review of the literature, Backholer et al. (2012) concluded that greater difficulties in daily activities were related to weight gain.

The results with regard to the psychological domain (i.e., self-esteem, body image, and negative and positive feelings) indicated that QoL declined with an increase in weight, which is consistent with previous studies (Kushner and Foster, 2000; Taylor et al., 2013). As highlighted by Wee et al. (2013), the stigma associated with obesity has an impact on various dimensions of QoL. The influence of obesity on psychological aspects, including self-esteem, body image, and emotional state, has been reported in the literature. These factors are influenced by the stigma associated with being overweight and obesity (Friedman et al., 2005; Thomas et al., 2010; Puhl and King, 2013). The stigma associated with obesity also appears to be an important factor in the decline of the social aspect of QoL of obese individuals (Wee et al., 2013). The social domain data revealed that self-evaluations of relationships and social support are negatively influenced by an increase in weight. These results can be explained by the marginalization of people who are overweight or obese. Different socio-epidemiological studies have highlighted the importance of social support for the well-being of individuals (Cummins et al., 2005; Poortinga, 2006). In fact, because of the stigma associated with obesity, people who are overweight or obese have difficulty maintaining social and professional relationships (Morris, 2007) and friendships (Ali et al., 2012). Social norms of body weight changed in Brazil during the last decades, closer to a European and North American body ideal, increasing the stigma toward obesity and influencing the impact of BMI on QoL.

The environmental aspects of QoL focus on physical safety with regard to access to transportation, leisure activities, and the availability of medico-social care. The present study corroborates the results in the literature, demonstrating a negative impact of elevated BMI on the perception of environmental characteristics (Poortinga, 2006). With regard to the social and environmental domains of QoL, the results did not show a linear relationship with BMI and may reflect a “dose-response effect.” According to McLaughlin and Hinyard (2014), when QoL is significantly reduced because of obesity, additional weight gain has little influence on the individual’s perceptions.

The present study also explored the presence of comorbidities in obese patients. We found that the number of illnesses was accentuated by increases in weight, regardless of gender. This result is consistent with recent studies on the relationship between obesity and other physical illnesses, which have reported that the number of pathologies presented by people who are overweight or obese increases with BMI (Dixon, 2010; Vucenik and Stains, 2012; Ojeda et al., 2014). The results also showed that women presented more illnesses than men. The difference between men and women in the number of health problems can be explained by the global disparity between genders with regard to health, suggesting that women are more prone to develop illnesses than men because of social, cultural, and economic reasons (World Health Organization, 2009).

The present study has some limitations. Similar to the majority of such studies (Yan et al., 2004), the groups included individuals with varying ages. Ideally, age would be included as an independent variable, with different groups according to age, but this would impact recruitment and study feasibility. Future studies that examine the link between obesity and QoL in young adults compared with older adults should be conducted to explore possible age factors. Additionally, BMI was calculated based on self-reported information, which, according to Stommel and Schoenborn (2009), can cause differences between the calculated BMI and real BMI. Future studies that use BMI should directly obtain the information that is necessary to calculate BMI. The lack of an overweight category can also be seen as a limitation of the study. However, previous studies showed small differences in QoL between this group and normal weight people (Hassan et al., 2003; Jia and Lubetkin, 2005). Another limitation of this study is the sample size. Nevertheless, the sample was sufficiently large to find group differences.

The present study is the first exploring the relationship between QoL and obesity in the adult population in Brazil. Only two studies exploring this relationship in Brazil have been published. Turco et al. (2013) compared obese and eutrophic adolescents using the PedsQL (Varni et al., 1999, Brazilian version by Klatchoian et al., 2008). They highlighted that obese adolescents had worse QoL compared to the eutrophic group across all domains evaluated. Horta et al. (2013) explored overweighed adult and elderly women with chronic non-communicable disease, using the WHOQOL-brief and showed that the “social relationship” domain is the one that contributed the most to QoL, followed by the “physical” domain. Our study replicated the results obtained by these two publications, extending the conclusions to another population (adult men and women).

This study is also one of the few exploring the relationship between QoL in a developing country. This relationship has already been extensively demonstrated in North American and European populations, but our results allow us to affirm that this relationship remains the same in a different social and cultural context. Additionally, the results suggest the possible influence of the sociocultural context on the relationship between weight, QoL and gender. Future studies that utilize a cross-cultural design may help to understand the contextual factors that are involved in the impact of obesity on QoL in relation to other variables, such as gender. We also suggest further explorations of the correlation between demographic information, such as socio-economic status, and the relationship between obesity/QoL, in particular for studies conducted in countries with higher inequality.

Our results indicate that an elevated BMI is associated with a reduction of overall QoL and reductions in various domains (i.e., physical, psychological, environmental, and social) in Brazilian men and women. Thus, the care and treatment of obese patients should be approached in a multidisciplinary way. In fact, obesity is a multifaceted condition impacting QoL in different ways, depending on the cultural context and, possibly, gender. The treatment of obesity is a difficult and complex process and, as recommended by the American National Institute of Health, a therapeutic approach emphasizing an increase in QoL on physical, psychological, environmental and social domains may facilitate loss of weight and a healthier lifestyle.

## Author Contributions

HS and JL-F conceived the study design. FP collected the data and drafted the manuscript. DM performed the data analysis. Critical revisions were contributed by DM and EB. All authors discussed the results, implications, and literature, and approved the final version of the manuscript for submission.

### Conflict of Interest Statement

The authors declare that the research was conducted in the absence of any commercial or financial relationships that could be construed as a potential conflict of interest.
